# Metaproteomics Provides Functional Insight into Activated Sludge Wastewater Treatment

**DOI:** 10.1371/journal.pone.0001778

**Published:** 2008-03-12

**Authors:** Paul Wilmes, Margaret Wexler, Philip L. Bond

**Affiliations:** 1 School of Environmental Sciences, University of East Anglia, Norwich, United Kingdom; 2 School of Biological Sciences, University of East Anglia, Norwich, United Kingdom; Portland State University, United States of America

## Abstract

**Background:**

Through identification of highly expressed proteins from a mixed culture activated sludge system this study provides functional evidence of microbial transformations important for enhanced biological phosphorus removal (EBPR).

**Methodology/Principal Findings:**

A laboratory-scale sequencing batch reactor was successfully operated for different levels of EBPR, removing around 25, 40 and 55 mg/l P. The microbial communities were dominated by the uncultured polyphosphate-accumulating organism “*Candidatus* Accumulibacter phosphatis”. When EBPR failed, the sludge was dominated by tetrad-forming α-*Proteobacteria*. Representative and reproducible 2D gel protein separations were obtained for all sludge samples. 638 protein spots were matched across gels generated from the phosphate removing sludges. 111 of these were excised and 46 proteins were identified using recently available sludge metagenomic sequences. Many of these closely match proteins from “*Candidatus* Accumulibacter phosphatis” and could be directly linked to the EBPR process. They included enzymes involved in energy generation, polyhydroxyalkanoate synthesis, glycolysis, gluconeogenesis, glycogen synthesis, glyoxylate/TCA cycle, fatty acid β oxidation, fatty acid synthesis and phosphate transport. Several proteins involved in cellular stress response were detected.

**Conclusions/Significance:**

Importantly, this study provides direct evidence linking the metabolic activities of *“Accumulibacter”* to the chemical transformations observed in EBPR. Finally, the results are discussed in relation to current EBPR metabolic models.

## Introduction

There is increasing interest to understand microbial community compositions and functions directly within their respective environments. Molecular analysis of environmental samples, mostly by analysis of 16S rRNA genes, has greatly improved our knowledge of the vast microbial diversity [1]. More recently, large metagenomic sequencing projects that analyse genomic DNA directly from environmental samples, are providing much detail of the genetic diversity and potential within selected environments, e.g. seawater samples [2], [3] and activated sludge [4]. A huge challenge is to couple this improved knowledge of microbial diversity with functional details of these microbial ecosystems. As most of the microbial biomass in environmental samples is presently unobtainable as isolated pure cultures, this effort requires *in situ* approaches.

In recent work, transcriptomic and proteomic analyses, traditionally used for study of pure cultures, are being applied to detect expression profiles and provide functional insight directly from mixed microbial environmental samples. Our recent work established for the first time that a proteomics approach could be successfully applied to examine protein expression in environmental samples such as activated sludge [5]. Since then there has been only a handful of studies describing mixed culture proteomics (termed metaproteomics) [6]. These include examination of protein expression profiles from an estuary transect [7], infant fecal samples [8], freshwater samples following exposure to heavy metals [9] and contaminated soil and groundwater [10]. Proteomic analysis of soil and water was used to determine microbial taxonomic groups in those environments [11], and differentially expressed proteins from bacterial communities following exposure to cadmium were detected [12]. Notably, a high-throughput proteomic study of acid mine biofilms has been performed [13], in which a large number of proteins (∼2,000) were identified; one novel protein was confirmed as a key component of energy conservation in that environment [13]. Consequently, despite the limited number of investigations, the metaproteomic approach has already highlighted its potential for providing functional insight into overall microbial ecosystem function [6].

Biological wastewater treatment plants (WWTPs) employing activated sludge represent the most widely used biotechnological process on Earth. The removal of organic carbon and other nutrients, mainly nitrogen and phosphorus (P), is essential to avoid the deterioration of receiving surface waters [14]. WWTPs can be engineered to enable enhanced biological phosphorus removal (EBPR). These WWTPs are characterised by an anaerobic treatment phase that precedes an aerobic phase. These systems select for particular bacteria, which accumulate large amounts of intracellular polyphosphate (polyP), causing the desired P removal during wastewater treatment. While EBPR is used globally with success, the systems do suffer intermittent periods of poor performance, and improvements of operation and performance are pressing in view of future constraints on the water cycle enhanced by global climate change.

There is great interest to understand the biochemistry of EBPR. However, most details of the process remain elusive, and this is partly because, in spite of many attempts, the polyphosphate accumulating organisms (PAOs) responsible for EBPR have not yet been isolated [15]. Nevertheless, based on the EBPR transformations and general bacterial metabolism, metabolic models have been derived to describe the energetic and substrate requirements. During the initial anaerobic phase, PAOs degrade stored polyP and glycogen, and synthesise polyhydroxyalkanoates (PHAs) from short chain volatile fatty acids (VFAs). In the subsequent aerobic stage, they store polyP and glycogen, and degrade PHAs. With the advent of molecular techniques, dominant PAOs in laboratory-scale EBPR systems are found to be members of the β-*Proteobacteria* and close relatives of *Rhodocyclus* spp. [16]–[19]. This group of PAOs are tentatively named “*Candidatus* Accumulibacter phosphatis” (herein described as *“A. phosphatis”*; [19]).

Although *“A. phosphatis”* remains uncultured, the phylogenetic identity of these dominant PAOs has recently provided opportunity for *in situ* investigations of microbial function. These studies have been many, and include use of MAR-FISH [20], and estimations of EBPR stoichiometry and kinetics [21], [22]. A recent major achievement has been the acquisition of EBPR metagenomic sequences [4]. These were obtained from two EBPR laboratory-scale reactors operated in Australia and the United States, henceforth referred to as the OZ and US sludge, respectively. Both reactors were enriched for *“A. phosphatis”* allowing García-Martín *et al.* [4] to subsequently assemble a composite *“A. phosphatis”* genome from the US metagenomic sequences and infer the metabolic pathways employed by *“A. phosphatis”* during EBPR. This provides much information on the genetic blueprint for EBPR, however, it does not provide direct functional information, speculated metabolisms still require verification, and system dynamics remain elusive.

Metaproteomics can be used to study protein expression from a complex system and provide direct evidence of metabolic and physiological activities. Recently, we used a proteomic approach through a combination of two-dimensional polyacrylamide gel electrophoresis (2D-PAGE) for quantitative protein detection and mass spectrometry-based protein identification [5]. The proteomic approach is now more feasible as the metagenomic sequences provide increased opportunity to identify proteins. In the present study, we compared protein expression in sludge with differing EBPR performance. We focused on identification of highly expressed proteins that would be central to the EBPR metabolism. Numerous proteins were detected that could be directly linked to EBPR metabolism and to the dominant PAO, *“A. phosphatis”*. Furthermore, we demonstrate the presence of highly expressed proteins whose activities have not been previously linked to EBPR and which, consequently, may need to be included in future metabolic models.

## Results and Discussion

### Generation of sludge with differing EBPR performance

A sequencing batch reactor (SBR) was operated for four different levels of EBPR over a period of more that 100 days, by alteration of the phosphorus levels (as phosphate) in the reactor feed. Three sludges with good EBPR performance were obtained. For these sludges, removal of phosphate-P from the influent was complete at 28.5, 42.4, and 55.2 mg/l, and this was stable for at least three sludge ages in each case. These sludges were termed EBPR_28_, EBPR_42_ and EBPR_55_, respectively (Table 1). Reactor cycle studies demonstrate that the three sludges carried out metabolic transformations typical of EBPR (Table 1, Fig. S1). This included the release of orthophosphate into the medium during the anaerobic phase followed by the concomitant uptake of the excreted orthophosphate during the aerobic phase. In contrast to the sludges above, a fourth sludge did not perform EBPR. When the SBR phosphate-P feed was increased to 70.7 mg/l the EBPR performance failed, and an average of 66.7 mg/l phosphate-P remained in the reactor effluent. This sludge was termed nEBPR_70_. Low levels of P transformations detected in the nEBPR_70_ sludge's cycle study were consistent with the absence of EBPR (Table 1 and Fig. S1D). The carbon transformations of the nEBPR_70_ were similar to those observed in EBPR sludges, with anaerobic PHA accumulation (Table 1), although anaerobic acetate uptake was not complete (Fig. S1D). This performance is characteristic of glycogen accumulating organisms (GAOs) that are implicated in EBPR failure in anaerobic/aerobic activated sludge systems [23].

**Table 1:** Summary of the EBPR transformations detected at various stages of the sequence batch reactor (SBR) performance.

	Sludge			
	EBPR_28_	EBPR_42_	EBPR_55_	nEBPR_70_
SBR feed phosphate-P (mg/l)	28.5	42.4	55.2	70.7
MLSS (g/l)	1.9 (0.14)	2.2 (0.63)	2.1 (0.3)	2.4 (0.06)
Biomass P content (%)	12.1	21.2	24.3	2.7
Anaerobic P release (mg/g MLSS)	64.0	60.6	84.2	8.8
Anaerobic PHA accumulation (mg/g MLSS)	56.1	36.5	52.3	40.4

Standard deviations in brackets.

### Microbial community analyses of the EBPR and nEBPR sludge

**Figure 1:**
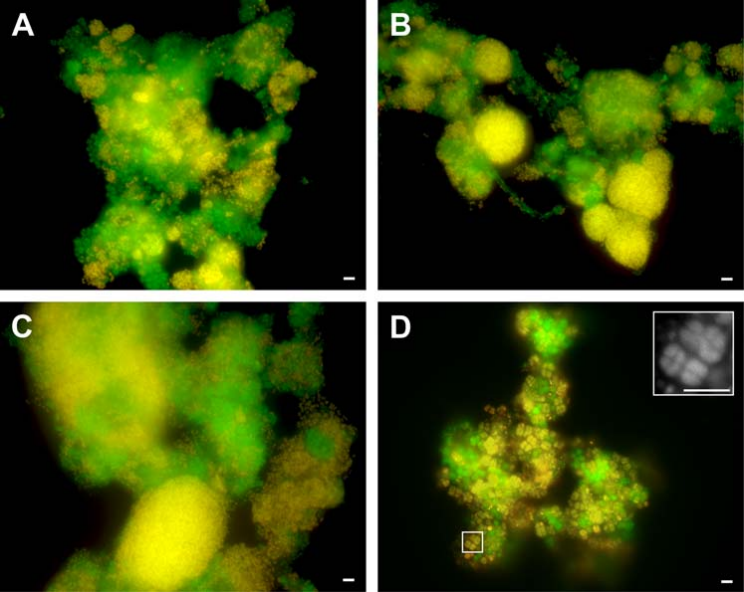
Representative FISH micrographs of the activated sludges analysed in this study. (A) EBPR_28_ sludge, (B) EBPR_42_ sludge, (C) EBPR_55 _sludge and (D) nEBPR_70_ sludge. Cells detected with probe EUBMIX only are green (A, B, C and D). Cells detected with both EUBMIX and PAO651 probes (A, B and C) and cells detected with both EUBMIX and ALF1b probes (D) are yellow-orange. Highlighted area in pane d corresponds to magnified region hybridised only with the ALF1b probe in the top right hand corner. Images taken under the different excitation wavelengths for CY3 and FITC were combined using Adobe Photoshop. Cells were observed under *x* 630 magnification, bars = 10 µm.

The sludge microbial communities were analysed by fluorescent *in situ* hybridisation (FISH). Most of the cells that stained positively with DAPI were identified as bacteria with the EUBMIX probes (98 %). The EBPR sludges, at each P increase level, were all dominated by organisms forming distinct coccoid clusters of cells (Fig. 1A,B&C). The EBPR_28_, EBPR_42_ and EBPR_55_ sludges gave positive signals for the β-*Proteobacteria* specific probe (63, 68 and 72 % of EUBMIX binding cells, respectively; Table S1) and the *“A. phosphatis”* specific probe PAO651 (61, 67 and 69 % of EUBMIX binding cells, respectively; Table S1). These results were similar to those found for sludges with similar P removal performances and biomass P contents [5], [18], [24], [25]. The number of cells belonging to both β-*Proteobacteria* and *“A. phosphatis”* were found to be significantly different in the three EBPR sludges (ANOVA, P<0.05) and, consequently, provide a causative link between the dominance of the *Rhodocyclus*-type PAO and P removal performance. High abundances of α-*Proteobacteria* were also observed in the EBPR_28_, EBPR_42_ and EBPR_55_ sludges, at 34, 29 and 25 %, respectively (Table S1). These consisted mainly of tetrad-arranged coccoid cells, as described previously [25]. Thus, the numbers of *Rhodocyclus*-type PAOs increased as the tetrad-forming α-*Proteobacteria* decreased. A possible explanation is that these two groups of organisms are in direct competition with one another as suggested previously [15].

In contrast to the EBPR sludges, the nEBPR_70_ sludge was dominated by the tetrad-arranged coccoid cells already observed in the EBPR sludges (Fig. 1D), and again identified as α-*Proteobacteria* (53 % of EUBMIX binding cells). The nEBPR_70_ sludge still revealed a rather high abundance of β-*Proteobacteria* (41 % of EUBMIX binding cells), but *“A. phosphatis”* was present in very low numbers (<1%; Table S1). The dominance of the tetrad-arranged α-*Proteobacteria* concurs with other investigations of anaerobic:aerobic non-P removing sludges [14], [26], [27]. Thus, our study again implicates this subphylum and cell type as GAOs that can dominate when EBPR fails.

### Metaproteomics of the EBPR sludges

#### 2D-PAGE analysis

**Figure 2:**
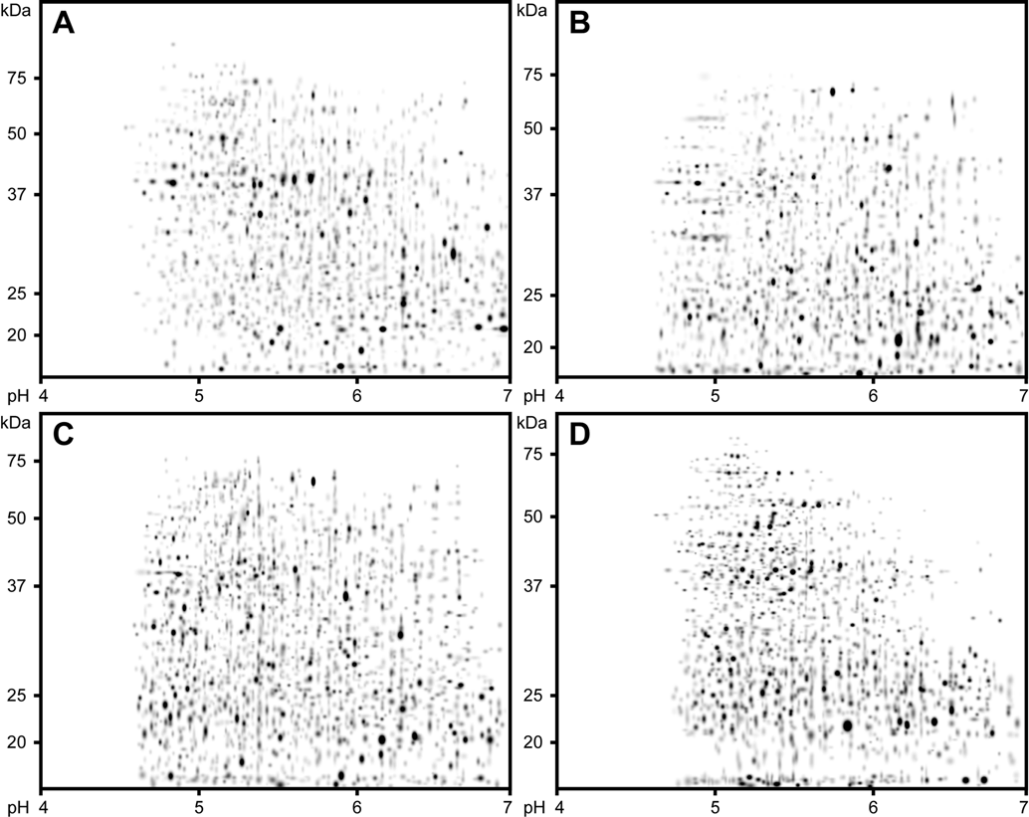
Representative 2D-PAGE separations of proteins extracted from (A) the EBPR_28_ sludge, (B) the EBPR_42_ sludge, (C) the EBPR_55 _sludge and (D) the nEBPR_70_ sludge. Approximate protein molecular mass ranges are provided on the left and isoelectric point ranges are provided on the bottom of the gel images.

**Figure 3:**
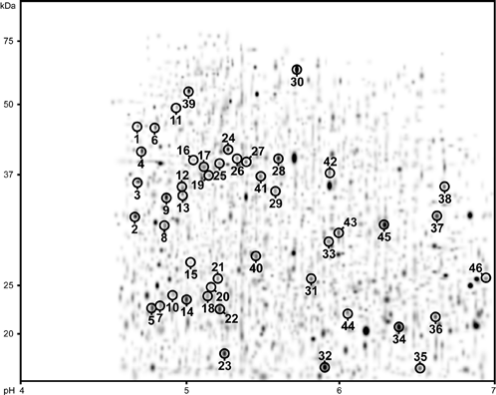
Master 2D-PAGE gel of the EBPR matchset with excised protein spots highlighted. Spot numbering corresponds to the numbering used in Table 2, and supporting information Table S3.

For each P increase level, 2D-PAGE separations of the proteins extracted from the four distinct sludges were carried out (Fig. 2), and from the gel separations metaproteomic maps were generated. Liquid isoelectric focussing prior to 2D-PAGE was employed to improve the gel separations. For each sludge highly reproducible metaproteomic maps were obtained, with >98 % of protein spots being matched within replicate gels (n = 3; Table S2). Additionally, the proteomic maps generated from the EBPR_28_, EBPR_42_ and EBPR_55_ sludges exhibited high similarity. Overall, a total of 638 spots were matched between all the gels for the EBPR sludges, and these conserved spots are represented on the “Master” gel (Fig. 3). The proteomic maps produced from nEBPR_70_ sludge samples were distinct compared to those generated from the EBPR sludges, and were analysed separately.

Each of the matched spots was quantified (by intensity and size) across the matchset of the three EBPR sludges (see Table S3 for quantitative comparison). The spots were then ranked according to their respective quantities in the EBPR_55 _gel set. 111 protein spots with the highest quantities were chosen for excision from a separate EBPR_55_ gel.

#### Mass Spectrometry (MS) analysis and protein identification

Excised protein spots for which positive identifications were obtained are highlighted on the 2D-PAGE master gel (Fig. 3). Details on the excised protein spots identified are listed in Table 2, and additional details are presented in Table S3.

**Table 2:** Identification and putative function of proteins excised from the EBPR_55_ 2D-PAGE gel and analysed by mass spectrometry (spot numbers refer to those in Fig. 3).

Spot no.	Description	Source^a^	Assignment^b^	% identity to *“A. phosphatis”* sequence^c^	Function
2, 5, 9, 27, 28	Poly(3-hydroxyalkanoate) synthetase	USP	other *“Accumulibacter”*	92	PHA synthesis
41	Acetyl-CoA acetyltransferase	USJ	other *“Accumulibacter”*	91	
		USP			
40	Enoyl-CoA hydratase	USJ	*“A. phosphatis”*	100	PHA synthesis and fatty acid β oxidation
		USP			
22	Acyl-CoA dehydrogenase	USP	other *“Accumulibacter”*	72	Fatty acid β oxidation
44	Acyl-CoA synthetase/AMP-(fatty) acid ligase	OZP	*“A. phosphatis”*	100	
		USJ			
		USP			
39	Biotin carboxylase	USP	other *“Accumulibacter”*	88	Fatty acid synthesis
7	Triosephosphate isomerase	USP	other *“Accumulibacter”*	93	Glycolysis (Embden-Meyerhof pathway)
33, 38	Phosphoenolpyruvate synthase	USJ	other *“Accumulibacter”*	92	Gluconeogenesis
		USP			
37	ADP-glucose pyrophosphorylase	USJ	other *“Accumulibacter”*	43	Glycogen synthesis
18	Hydroxypyruvate isomerase	USP	other *“Accumulibacter”*	47	Glyoxylate/tricarboxylic acid metabolism
20	Malate synthase	USP	other *“Accumulibacter”*	92	
21	Succinate dehydrogenase/fumarate reductase	OZP	*“A. phosphatis”*	100	
		USJ			
		USP			
43	Citrate synthase	USJ	other *“Accumulibacter”*	95	
36	ABC-type phosphate transport system, periplasmic component	USP	other *“Accumulibacter”*	99	Phosphate transport
1, 4, 8, 11, 14	F_0_F_1_-type ATP synthase, β subunit	USP	other *“Accumulibacter”*	95	ATP regeneration
6, 12	F_0_F_1_-type ATP synthase, β subunit	OZP	*“A. phosphatis”*	100	
26	Uncharacterised NAD(FAD)-dependent dehydrogenase	USJ	other *“Accumulibacter”*	62	
23	Peroxiredoxin	OZP	*“A. phosphatis”*	100	Oxidative stress response
		USJ			
		USP			
32	Peroxiredoxin	USP	other *“Accumulibacter”*	/	
30	Thiol-disulfide isomerase and thioredoxin	OZP	*“A. phosphatis”*	100	Protein folding
17	Chaperonin GroEL, HSP60 family	USP	other *“Accumulibacter”*	95	
19	Chaperone, HSP90 family	USJ	other *“Accumulibacter”*	90	
29	Chaperone, HSP90 family	USP	other *“Accumulibacter”*	90	
3	Outer membrane protein and related peptidoglycan-associated (lipo)proteins	USJ	other *“Accumulibacter”*	42	Membrane protein
10	Aspartate/tyrosine/aromatic aminotransferase	USJ	other *“Accumulibacter”*	81	Amino acid metabolism
13, 16	GTPase-translation elongation factor	USP	other *“Accumulibacter”*	99	Translation
24, 25	GTPase-translation elongation factor	OZP	*“A. phosphatis”*	100	
		USJ			
		USP			
45	Glutamyl- and glutaminyl-tRNA synthetase	OZP	*“A. phosphatis”*	100	
		USJ			
		USP			
15	Transcription elongation factor	USP	other *“Accumulibacter”*	93	Transcription
42	Topoisomerase IA	OZP	other *“Accumulibacter”*	53	
31	2-keto-4-pentenoate hydratase	USJ	other *“Accumulibacter”*	82	Catechol pathway
		USP			
34	Protein of unknown function	USP	*“A. phosphatis”*	100	Unknown function
35, 36	Protein of unknown function	USP	other *“Accumulibacter”*	83	
46	Protein of unknown function	USJ	other *“Accumulibacter”*	/	
		USP			

a Abbreviations: OZP: OZ sludge, Phrap assembly; USJ: US sludge, Jazz assembly; USP: US sludge, Phrap assembly

b Assignment is based on the IMG/M binning of the genomic sequences. Sequences were binned as *“A. phosphatis”* as determined by US/OZ overlap [4]. Sequences assigned to other *“Accumulibacter”* contains sequences that belong to the genus *“Accumulibacter”* but not the species *“A. phosphatis”*

c The % nucleotide identity of the MASCOT matched sequence against the *“A. phosphatis”* sequence.

Initially, 38 of the 111 chosen proteins were positively identified from their respective peptide mass fingerprints (by MALDI-ToF MS/MASCOT) searched against the EBPR sludge metagenomic databases (numbered 1–38; Table 2; see Materials and Methods for details on the databases). Protein digests, which did not result in positive identifications using MALDI-ToF MS/MASCOT and for which clear MS spectra had been obtained, were further analysed using Q-ToF MS/MS. The resulting peaklists were again searched using MASCOT. A further 8 protein spots were identified (numbered 39–46; Table 2). Overall, 41 % of the excised protein spots were identified. The rather low identification ratio may be due to strain variation between the sludges from which the metagenomic data were obtained (OZ and US sludges) and those from which the proteins were isolated (our EBPR_55_ sludge), since the chosen stringent protein identification strategy relies on exact peptide matches. Nonetheless, the availability of metagenomic sequences allows for rapid protein identification compared to previous investigations relying on cost- and time-intensive *de novo* peptide sequencing [5].

A number of proteins were identified several times from different individual spots, e.g. F_0_F_1_-type ATP synthase, beta subunit (spots 1, 4, 6, 8, 11, 12 and 14). Highly abundant proteins may swamp the 2D-PAGE gels, leading to multiple detection. This artefact of 2D-PAGE has been noted in pure culture studies and may be due to several reasons, including strain variation, differential protein processing, posttranslational modifications, and protein degradation [28], [29]. Activated sludge is especially rich in lipases and proteases [30] and, despite inclusion of protease inhibitors in the protein extraction buffers (see Materials and Methods), some protease activity may have been retained in the crude protein extracts. In total, 33 unique proteins were detected after excluding redundant identifications.

Protein identifications were deduced by matching the MS peaklist data to the metagenomic gene sequences using the MASCOT algorithm. The subsequent gene and protein functions were derived from the IMG/M annotation of the metagenome data [31]. Consequently, the putative protein functions are discussed here in relation to current EBPR metabolic models, and gene synteny information is included when relevant. Importantly, the present study highlights details of metabolic pathways active during EBPR, it reveals pathways previously not considered in metabolic models, and it provides direction for future investigations defining enzyme activities and regulatory events.

### Suggested protein functions in relation to EBPR

#### Fatty acid oxidation and PHA synthesis

**Figure 4:**
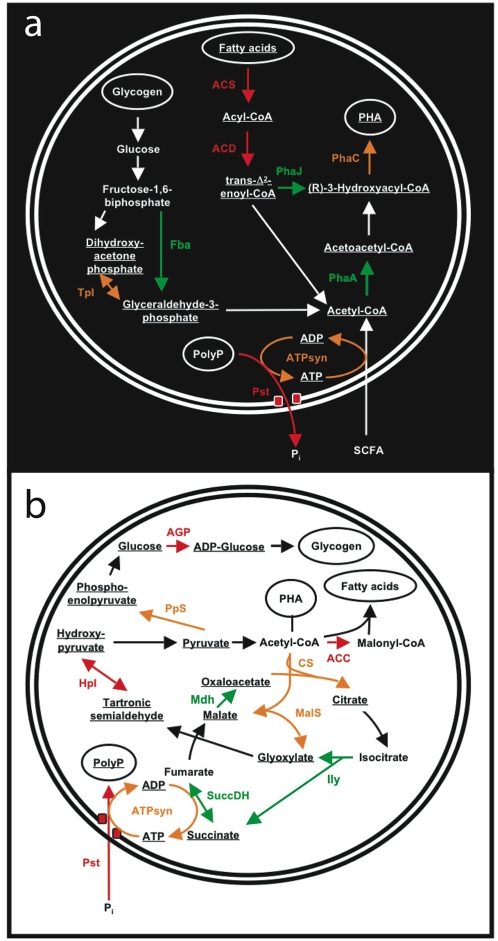
Proposed metabolic model for the (A) anaerobic and (B) aerobic phase of EBPR inferred from the proteomic data. Identified proteins catalysing individual reactions are highlighted in green [best MASCOT metagenomic sequence match located on a scaffold source binned as *“A. phosphatis”*, i.e. strong association with the *“A. phosphatis”* composite genome], orange [best MASCOT sequence match located on a scaffold source binned as “other *Accumulibacter”* for which a strong BLAST hit (>90 % identity) was obtained with a sequence binned as *“A. phosphatis”*, i.e. medium strong association with the *“A. phosphatis”* composite genome], and red [best MASCOT sequence match located on a scaffold source binned as “other *Accumulibacter”* for which a weak BLAST hit (<90 % identity) was obtained with a sequence binned as *“A. phosphatis”*, i.e. weak association with the *“A. phosphatis”* composite genome]. Not all intermediate metabolites are shown. Abbreviations: ACC, acetyl-CoA carboxylase; ACD, acyl-CoA dehydrogenase; ACS, acyl-CoA synthetase; AGP, ADP-glucose pyrophosphorylase; ATPsyn, F_0_F_1_-type ATP synthase; CSY, citrate synthase; Fba, fructose bisphosphate aldolase; HpI, hydroxypyruvate isomerase; Ily, isocitrate lyase; Mdh, malate dehydrogenase; MalS, malate synthase; NADH, uncharacterised NAD(FAD)-dependent dehydrogenase; PhaA, acetyl-CoA acetyltransferase; PhaC, poly(3-hydroxyalkanoate) synthetase; PhaJ, enoyl-CoA hydratase; PpS, phosphoenolpyruvate synthase; Pst, ABC-type phosphate transport system; SCFA, short chain fatty acids; SuccDH, succinate dehydrogenase; TpI, triosephosphate isomerase.

The detected proteins along with possible functions are listed in Table 2. Several proteins putatively involved in PHA synthesis and fatty acid oxidation were highly expressed. These included acetyl-CoA acetyltransferase (PhaA; spot 41), which is involved in the formation of acetoacetyl-CoA, the first step of PHA synthesis, and poly (3-hydroxyalkanoate) synthetase (PhaC; spots 2, 5, 9, 27 and 28). The activity of PhaC links *(R)*-3-hydroxyacyl-CoA to an existing PHA molecule, the last step in the formation of PHA (Fig. 4a). These transformations would be an integral part of anaerobic EBPR metabolism.

Enoyl-CoA hydratase (PhaJ) was also detected (spot 40). The activity of this enzyme may directly link PHA formation to fatty acid β oxidation (Fig. 4a) [32]. Interestingly, all of the EBPR metagenomic contigs that contain *phaJ* also contain adjacent genes involved in PHA synthesis. From pure culture studies, the expression of *phaJ* is co-regulated with other PHA synthesis genes and in certain bacterial species *phaJ* is part of a PHA synthesis operon [33]. However, based on nucleotide spacing, the genes described here are unlikely to represent an operon. Overall, three out of the five key enzymes involved in PHA formation [32] were identified from the reactor sludge and are present on contigs and scaffolds linked to *“A. phosphatis”*. Consequently, our evidence of protein expression implicates PHA synthesis by *“A. phosphatis”* in the context of EBPR (Fig. 4a).

In addition to PhaJ, other proteins involved in fatty acid β oxidation were identified. Protein spots 22 and 44 were identified as acyl-CoA dehydrogenase and acyl-coenzyme A synthetase/AMP-(fatty) acid ligase respectively. These proteins are involved in the activation and the initial step of fatty acid β oxidation (Fig. 4a). Furthermore, protein spot 39 was identified as a biotin carboxylase assigned to *“A. phosphatis”* which forms part of the acetyl-CoA carboxylase complex. That complex catalyses the first committed and rate-limiting step of fatty acid synthesis. Furthermore, one of the *“A. phosphatis”* contigs that contains biotin carboxylase also contains a putative acyl dehydratase, an enzyme characteristic of aerobic fatty acid biosynthesis.

We hypothesise that fatty acid metabolism plays an important role in EBPR biochemistry beyond that of lipid metabolism for cell membranes (Fig. 4). One possibility is that it acts as an additional storage molecule in PAOs and fulfils a similar role compared to glycogen as suggested in previous metabolic models, e.g. [14]. Fatty acid, if accumulated in the aerobic phase, could provide more reducing equivalents during the anaerobic phase, in comparison to glycogen. Neutral lipid storage molecules are widespread in eukaryotes, but have only been reported in relatively few bacteria including some actinomycetes and *Acinetobacter* species [34]. We postulate that anaerobic oxidation of stored fatty acids is important for contributing reducing equivalents. Furthermore, the utilisation of exogenous fatty acids may be relevant in full-scale EBPR systems that are not fed acetic acid based synthetic feed. In laboratory-scale reactors, propionate has been found to be a more favourable substrate for EBPR compared to acetate [22] and the provision of longer volatile fatty acids would require less energy expenditure for the accumulation of intracellular macromolecules (PHA and fatty acids). Consequently, fatty acid accumulation and degradation may have direct ramifications on the engineering of EBPR wastewater treatment systems. Finally, it should be noted that in some species PhaJ catalyses the formation of the PHB precursor (*R*)-3 hydroxyacyl-CoA *via* the intermediate crotonyl-CoA, from acetyl-CoA precursors [35]. Thus, another possible role for PhaJ detected in our study is PHB synthesis *via* this pathway. Overall, the exact role of fatty acid metabolism in EBPR warrants further investigation.

#### Glycogen degradation and synthesis

One contentious issue regarding details of the EBPR metabolic model has been the nature of the glycolytic pathway used by PAOs in the anaerobic degradation of glycogen. Protein spot 7 was identified as triosephosphate isomerase. This supports previous suggestions that the Embden-Meyerhof is the key glycolytic pathway in EBPR [4]. However, this disagrees with metabolic evidence suggesting the Entner-Doudoroff pathway is used [36]. Further evidence of key enzyme activity is required to determine the glycolytic pathway used by PAOs. In a recent unpublished investigation of another EBPR sludge we detected high expression of fructose bisphosphate aldolase, further supporting the Embden-Meyerhof pathway as the suggested glycolytic pathway. In regard to glycogen synthesis, protein spots 33 and 38 revealed phosphoenolpyruvate synthase and protein spot 37 was identified as ADP-glucose pyrophosphorylase. Thus, key enzymes involved in carbohydrate degradation and storage were identified (Fig. 4).

#### The glyoxylate/TCA cycles

Our proteomic analysis identified hydroxypyruvate isomerase (spot 18) which exclusively catalyses the reversible isomerisation between hydroxypyruvate and tartonate semialdehyde (Fig. 4b). In bacteria the expression of this gene is induced by the presence of glyoxylate. Three other enzymes linked to the glyoxylate/tricarboxylic acid (TCA) cycle were highly expressed including malate synthase (MalS; spot 20) which catalyses the condensation of acetyl-CoA and glyoxylate with the formation of malate and CoA (Fig. 4b). Spot 21 revealed the Fe-S protein subunit of succinate dehydrogenase/fumarate reductase (SuccDH). On the corresponding *“A. phosphatis”* scaffold genes coding for other subunits of the enzyme preceded the gene encoding the Fe-S subunit. SuccDH catalyses the reversible conversion of succinate to fumarate, as part of the TCA or the glyoxylate cycles (Fig. 4b). In further unpublished work examining differential expression between the anaerobic and aerobic phases of EBPR, we detected increased expression of other glyoxylate cycle proteins, malate dehydrogenase and isocitrate lyase, in the aerobic phase of EBPR (Fig. 4b). Other enzymes of the TCA/glyoxylate cycle that are located on the *“A. phosphatis”* composite genome mediate the cyclic reactions, e.g. citrate synthase (protein spot 43). Taking these results into consideration, it is clear that the glyoxylate cycle is active in EBPR, and likely this is more so in the aerobic phase (Fig. 4b).

As alluded to earlier, the source of reducing power during the anaerobic PHA synthesis has long been a point of interest. This was originally proposed through oxidation of acetate via the TCA cycle [37]–[39] or through degradation of intracellular glycogen [40]. The glyoxylate cycle has also recently been proposed for producing reducing equivalents in the anaerobic phase [41], [42], possibly in conjunction with a novel cytochrome [4]. Those previous suggestions were based on model bacterial pathways and the presence of genes on the metagenome. Our study provides evidence of high expression of proteins involved in the glyoxylate cycle, for the first time directly implicating its importance in EBPR metabolism. The glyoxylate shunt allows the production of reducing equivalents (for subsequent energy conservation) without the release of carbon dioxide in the conversion of isocitrate to succinate, in contrast to the reactions of the TCA cycle. Consequently, we argue that the operation of the glyoxylate shunt is more critical in the aerobic phase, where balancing carbon substrate utilisation for energy conservation and storage molecule (glycogen) synthesis is essential (Fig. 4b). This idea is supported by our recent detection of differential expression of other glyoxylate enzymes (unpublished data).

#### Phosphate transport and bioenergetics

Two separate proteins were identified from protein spot 36. A hypothetical protein, and a periplasmic component of an ATP binding cassette (ABC)-type phosphate specific transport system (Pst). The gene producing the best MASCOT match was located on a contig assigned to “other *Accumulibacter”* that contained four other genes encoding the Pst system, namely three permease components and the ATPase component. In other bacteria the Pst transport system couples the hydrolysis of ATP to the translocation of phosphate across the inner membrane [43]. These are typically high affinity transporters that are only expressed during sub micromolar concentrations of extracellular phosphate and the specificity of Pst systems is for protonated phosphate species but not metal phosphates. Consequently, PAOs may use active transport for uptake and/or release of phosphate. It is proposed that the activity of this high affinity phosphate transport system may be especially pronounced in the later stages of the aerobic phase when P concentrations are limited (Fig. 4b), and that a low affinity inorganic phosphate transporter (Pit) is the more active system during the other stages [4]. Another possibility is that the Pst system detected here is active throughout the different stages of EBPR (Fig. 4) and the anaerobic phosphate efflux could mediate the production of ATP. Such activity would have direct ramifications for EBPR bioenergetics and biochemical characterisation of the PAO phosphate transport systems is required to reveal the exact physiological details.

The protein identified from the largest number of individual spots (7) was the β subunit of F_0_F_1_-type ATP synthase. The β subunit of F_0_F_1_-type ATP synthase is a non-membrane spanning protein and expression from *“A. phosphatis”* and “other *Accumulibacter*” were detected. All the genes coding for the F_1_ unit of ATP synthase were contained on a single metagenome scaffold, i.e. γ, α and δ subunits, and subunit β. Protein spot 26 was identified as an uncharacterised NAD(FAD)-dependent dehydrogenase possibly forming part of the electron transport chain within *“A. phosphatis”*. Consequently, both detected proteins may be involved in ATP regeneration within *“A. phosphatis”* and, as such, fulfil an essential role in the EBPR metabolic model (Fig. 4).

#### Stress response and other proteins

A number of stress response proteins were highly expressed in the EBPR sludge. Protein spots 23 and 32 were identified as separate peroxiredoxins, which protect cells against reactive oxygen species. Protein spot 30 revealed another oxidative stress induced protein, thiol-disulfide isomerase, thioredoxin. Thioredoxins are responsible for maintaining disulfide bonds within cytoplasmic proteins in a reduced state and, hence, are required for proper folding of proteins. Other proteins directly involved in protein folding were also detected, including molecular chaperone proteins. Proteins associated with cellular stress response mechanisms actually represented the largest fraction of proteins identified in this study. It is reasonable to expect that cells within the EBPR biomass would experience stress such as large changes in redox potential in short periods of time. Consequently, cells able to maintain protein function in the alternating anaerobic:aerobic sludge cycling are favoured.

Numerous housekeeping proteins not specific to EBPR were detected (Table 2). Only few 2D-PAGE gel spots were identified as proteins of unknown functions (protein spots 34, 35 and 46). This is in contrast to the metaproteomic investigation of the acid mine drainage biofilm in which the largest fraction of detected proteins were of unknown function [13].

#### Strain resolved community proteomics

It is evident that all the proteins' best MASCOT hits (which are a function of unique and shared peptide masses) were against sequences binned as *“Accumulibacter”* species but not specifically binned as *“A. phosphatis”* (Table 2). The logical explanation is that we are detecting proteins highly expressed by the dominating PAOs in our reactor and that these are distinct but closely related to the dominant *“A. phosphatis”* strain assembled by García-Martín *et al.* [4]. Overall, the vast majority of best MASCOT hits were obtained against the Phrap assembly of the US sludge (Table 2), followed by the Jazz assembly of the US sludge and, lastly, by the Phrap assembly of the OZ sludge. Consequently, the metagenomic sequences obtained from the US sludge better reflect the genetic make-up of the EBPR sludges described in this study.

Numerous other *“A. phosphatis”* strains (4 % divergent at the nucleotide level from the dominant strain) and *“Accumulibacter”* species (15 % divergent at the nucleotide level from the dominant *“A. phosphatis”* strain) were present in the US and OZ sludges (Kunin, V. and Hugenholtz, P.; unpublished). Furthermore, previous work has revealed extensive diversity among *“Accumulibacter”*-related organisms [44]. While our quantitative FISH analysis revealed dominance of *“Accumulibacter”*-type organisms, strain resolution was not obtained. Thus, it seems likely an *“Accumulibacter”* strain different from the assembled *“A. phosphatis”* dominated our reactor, and would have contributed to limiting protein identifications based on the MS hits against metagenome sequence. Consequently, the present study highlights the requirement for strain-resolved community proteomics in environmental microbiology research [45]. The use of advanced instrumentation, e.g. liquid chromatography electrospray two-dimensional linear ion trap mass spectrometry in conjunction with the Orbitrap detector [46], will allow the differentiation of highly expressed proteins at the *“A. phosphatis”* strain and/or *“Accumulibacter”* species level.

### The metaproteomic approach

The present study highlights the opportunity and power of applying proteomics to mixed culture systems for which metagenomic sequences are available. This is particularly applicable in systems that are well characterised with respect to biochemical transformations and have rather limited diversity. The classical 2D-PAGE proteomic approach was used in this study. In comparison, multidimensional liquid chromatography coupled to MS, such as that recently used to detect proteomes from a mixed culture biofilm in an acid drainage solution [13], has potential for much higher throughput for protein identifications. However, the 2D-PAGE approach retains an advantage with regard to protein quantification since protein spot intensities and sizes on 2D-PAGE gels are a better reflection of protein abundance compared to abundances inferred from peptide MS data alone. Taking advantage of this characteristic of 2D-PAGE gels enabled us to focus on highly expressed proteins, and to monitor protein expression that increased with increasing P removal performance.

Numerous proteins that could be directly linked to the investigated metabolic mixed-culture process of EBPR were identified. The discovery of these functional enzymes is evidence for the described biochemical processes of EBPR metabolism. In addition, novel suggestions are made relating to PAO metabolism. These include the involvement of fatty acid metabolism and the glyoxylate shunt. Thus, we highlight potentially important functions and metabolic pathway details that have been overlooked in other EBPR studies. Additionally, this work provides important direction for future studies. Particular proteins detected here could be the focus of investigations for biochemical characterisation attempts to verify function, to examine regulatory details of expression, and to measure specific enzyme activities in full-scale EBPR systems.

## Materials and Methods

### Sequencing batch reactor operation and sampling

A laboratory-scale SBR with alternating anaerobic/aerobic phases was operated as described previously [5], [25]. Briefly, the reactor had a working volume of 2 l, and was operated on a 6 hr cycle consisting of a 120 min anaerobic phase, a 210 min aerobic phase and a 30 min settling/decant phase. Initially, the reactor was operated for approximately 1.5 months until stable EBPR performance, removing around 25 mg/l, was obtained. At this time-point (day of operation 0) an intensive sampling routine was started. This entailed measuring PO_4_-P in the influent (feed) and in the reactor at the end of the aerobic phase (t = 330 min) each day for a total of 102 days. On the tenth day of reactor operation, a cycle study was carried out. This involved taking samples for PO_4_-P, acetate, polyhydroxyalkanoate (PHA), mixed liquor suspended solids (MLSS) and total P analyses. At this time-point, the activated sludge within the reactor was completely removing 28.5 mg/l of PO_4_-P (no phosphate in the reactor at the end of the aerobic phase) and this constituted the EBPR_28 _sludge described in this study. On the 17^th^ day of operation, the PO_4_-P concentration in the feed was increased to around 40 mg/l. A complete cycle study was carried out on day 49 and this sludge was completely removing 42.4 mg/l PO_4_-P. This constituted the EBPR_42 _sludge described in this study. On day 57 the PO_4_-P concentration in the feed was increased to around 55 mg/l and a cycle study was carried out on day 71. The sludge was completely removing 55.2 mg/l PO_4_-P and, consequently, this constituted the EBPR_55 _sludge described in this study. On the 78^th^ day of reactor operation, the PO_4_-P concentration in the feed was increased to around 70 mg/l. Following this increase, the reactor's P removal performance started to fluctuate with 57.6 mg/l PO_4_-P present at the end of the aerobic phase on day 82. However, the reactor did regain its P removal performance on day 86. From day 92 onwards, the reactor gradually lost its EBPR performance with complete loss of P removal performance after 7 days, i.e. 1 sludge age. On day 99, another cycle study was carried out. 59.6 mg/l of extracellular PO_4_-P remained in the reactor at the end of the aerobic phase with 70.7 mg/l of PO_4_-P in the feed. This sludge constituted the nEBPR_70 _sludge described in this study.

### Chemical analyses

Phosphate P, acetate, mixed liquor suspended solids (MLSS) and total P were analysed as described earlier [5]. Polyhydroxyalkanoates (PHAs) were quantified following acid methanolysis by gas chromatography [16] with modifications described in the Supporting Material S1.

### 16S rRNA FISH with phylogenetic probes

Sampling, cell fixation, hybridisation and image processing were carried out as reported earlier [5]. Samples were taken of each of the analysed sludges (EBPR_28_, EBPR_42_, EBPR_55_ and nEBPR_70_) at the end of the aerobic phase. A range of broad and specific probes were employed and these were obtained from MWG Biotech (Ebersberg, Germany). Oligonucleotide probes ALF1b [47], BET42a [47], GAM42a [47], HGC69a [48], CF319a [47], GAOQ431 [49] and PAO651 [18] were labelled with the sulfoindocyanide dye Cy3 and EUBMIX [50] probes were labelled with fluorescein isothiocyanate (FITC). uBET and uGAM were unlabeled [51]. Single factor analysis of variance (ANOVA) was used to evaluate quantitative differences of FISH detected cells between the sludges.

### Protein extraction, purification and resuspension

100 ml samples were taken for each analysed sludge (EBPR_28_, EBPR_42_, EBPR_55_ and nEBPR_70_) at the end of the aerobic phase (t = 330 min). Protein extractions and purifications were carried out as described earlier [5], [25]. Following precipitation the protein pellets were resuspended in a resuspension buffer. The resuspension buffer consisted of 7 M urea, 2 M thiourea, 4 % (w/v) CHAPS, 40 mM Tris/1 mM EDTA, 50 mM dithiothreotol (DTT), 25 mM Pefabloc SC, 2 mM Pefabloc Protector (Roche, Welwyn Garden City, UK) and 1 % (v/v) ZOOM Carrier Ampholytes pH 3–10 (Invitrogen, Paisley, UK).

### Liquid isoelectric focussing

Liquid isoelectric focusing was carried out in a ZOOM IEF Fractionator (Invitrogen) according to the manufacturer's instructions. 2.2 mg of total protein were fractionated in each run. The pH 5.4 and 6.2 ZOOM Disks were excluded from the assembly to obtain a single pH 4.6–7.0 fraction. The liquid fractionation was carried out according to the following conditions: 100 V for 20 min, 200 V for 80 min and 600 V for 140 min. After fractionation, the different fractions were removed from the fractionator. Immobilised pH gradient (IPG) buffer pH 4–7 (Amersham Biosciences–GE Healthcare, Chalfont St. Gilles, UK) was added to the pH 4.6–7.0 fraction to obtain a final concentration of around 2 % (v/v) carrier ampholytes. 2-D SDS-PAGE standards (Bio-Rad, Bath, UK) were mixed into the pH 4.6–7.0 fraction according to the manufacturer's instructions.

### 2D-PAGE

The prepared pH 4.6–7.0 fractions were used to rehydrate 24 cm pH 4–7 IPG strips (Immobiline DryStrips, Amersham Biosciences–GE Healthcare) in an Immobiline DryStrip Reswelling Tray (Amersham Biosciences–GE Healthcare) for 16 hrs. For first-dimension separation, the strips were placed in an IPGphor ceramic manifold, covered with Plusone DryStrip cover fluid and focused for 100000 volt-hours in an Ettan IPGphor II isoelectric focusing system (Amersham Biosciences–GE Healthcare). The strips were then equilibrated [52] and applied to 14 % (v/v) Duracryl (Genomic Solutions, Huntingdon, UK) gels. Precision Plus Protein Standard Plugs (Bio-Rad) were layered onto the gels according to the manufacturer's instructions. Second dimension separation was carried out at 500 V in an Ettan DALT*six* electrophoresis system (Amersham Biosciences-GE Healthcare). The gels were stained overnight with SyproRuby (Bio-Rad) and scanned using a Molecular Imager FX (Bio-Rad). Triplicate 2D-PAGE separations were generated for each sludge sample.

### *In silico* analysis of 2D-PAGE gels

The acquired gel images were processed and analysed using PDQuest, version 7.3.0 (Bio-Rad). The EBPR_28_, EBPR_42_ and EBPR_55_ replicate gels were placed within the same matchset, termed the EBPR matchset. Since the nEBPR_70_ replicate gels were too dissimilar compared to the EBPR gels, they were placed in a separate matchset, i.e. the nEBPR matchset. Automated and manual spot detection and matching was performed, as well as spot densities determined for quantification. From the EBPR matchset a consensus gel (termed the “Master” gel) was produced.

### Spot excision

Following *in silico* analysis of the 2D-PAGE gels generated using PDQuest, spots were chosen for identification. The spots in the EBPR matchset were ranked according to their spot quantity as determined by PDQuest analysis. Protein spots that were highly expressed on the EBPR_55_ replicate group were chosen for excision. The chosen spots were also present on the EBPR_28_ and EBPR_42_ gels. These were excised from an EBPR_55_ 2D-PAGE gel using an Investigator Pro Pic (Genomic Solutions) spot-picking robot and placed in a 96 well plate prior to further processing.

### Matrix assisted laser desorption ionisation time-of-flight (MALDI-ToF) mass spectrometry (MS), Quadrupole-ToF (Q-ToF) MS/MS and protein identification

The excised spots were processed and digested as described earlier [5]. Details on mass spectrometry analyses and the protein identification strategy are provided in the Supporting Material file S1. Briefly, the digested protein samples were analysed on a Bruker UltraFlex MALDI-ToF/ToF mass spectrometer (Bruker Daltonics Ltd., Coventry, UK). Samples were further analysed using a Q-ToF-2 mass spectrometer (Micromass, Elstree, UK). The resulting peptide mass fingerprints were searched against the three metagenomic databases (OZ sludge, Phrap assembly; US sludge, Phrap assembly; US sludge, Jazz assembly) [4] using the MASCOT search tool (http://www.matrixscience.com). Detailed information on the metagenomic sequences of the best MASCOT hits were retrieved using the integrated microbial genomes with microbiome samples (IMG/M) system (experimental version; 1 September 2006, http://www.jgi.doe.gov/) [31]. For contigs and scaffolds of interest, Genbank files were generated using IMG/M and analysed using Artemis release 8 [53].

## Supporting Information

Material S1 Supporting material and methods
(0.10 MB DOC)

Table S1 Results of the quantitative FISH analysis (standard deviations in brackets).
(0.03 MB DOC)

Table S2 Summary of the 2D-PAGE analysis of the EBPR matchset (standard deviations in brackets). Indicating numbers of spots detected on individual gels and those matched across the replicate gel sets (n = 3).
(0.02 MB DOC)

Table S3 Protein identification results obtained using MALDI-ToF MS (spot numbers 1–39), Q-ToF MS/MS (spot numbers 40–46) and MASCOT including additional information (spot numbers refer to those in Fig. 3).
(0.88 MB DOC)

Figure S1 (4.07 MB TIF)
