# Supplementary material for: Metaproteomics Provides Functional Insight into Activated Sludge Wastewater Treatment
Source: PLoS One. 2008 Mar 12;3(3):e1778. doi: 10.1371/journal.pone.0001778 (PMC2289847; doi:10.1371/journal.pone.0001778)
Supplement: Material S1 — Supporting material and methods (0.10 MB DOC) [file pone.0001778.s001.doc]

**Supporting Materials and Methods**

**Chemical analyses.** Phosphate P, MLSS and total P were analysed as described earlier (Wilmes and Bond, 2004). For each cycle study that was carried out, samples for PO_4_-P analysis were taken every 15 min during one discrete cycle. Samples for mixed liquor suspended solids (MLSS) and total phosphate (tP) analyses were taken at the beginning of the cycle (t = 0 min). For acetate quantification, 10 ml sludge samples were taken every 15 min during the anaerobic phase of the reactor cycle. Each sample was immediately filtered (0.2 μm cellulose nitrate filter; Sartorius, Epsom, UK) and filtrates were placed at - 20 ^o^C before analysis. On the day of analysis, the samples were thawed, then filtered using a Millex-GP PES 0.22 μm filter (Millipore, Watford, UK) and with OnGuard II sample pre-treatment cartridges Ag, H and P (Dionex, Leeds, UK). The extracellular acetate levels were determined using a Dionex ICS2000 ion chromatograph (suppressed chromatography) with an AS18 separating column, an AG18 guard column and an EG40 eluent generator. The quantifications were carried out in triplicate for each sample. For PHA quantification, four replicate 20 ml SBR sludge samples were taken at the beginning of the anaerobic phase (t = 0 min), at the end of the anaerobic phase (t = 120 min) and at the end of the aerobic phase (t = 330 min). These were centrifuged at 4500 rpm (4 °C) for 5 min. The supernatants were decanted and the individual pellets were frozen at – 20 °C. The frozen pellets were transferred to 12 ml crimp top vials (Fisher) and lyophilised overnight. 2 ml of acidified [3 % (v/v) sulphuric acid] methanol and 2 ml of chloroform were added to the lyophilised sludge pellets. The vials were sealed using a silicone/Teflon seal (Fisher) and the mixtures digested for 20 hours in an oven at 104 °C. After cooling, 1 ml of water was added to each digestion and this was shaken for 10 min. The chloroform phase was allowed to settle to the bottom of the tube and this was then extracted for gas chromatography (GC). GC analysis was performed on an Agilent 6890 GC (with 6890 auto-injector) coupled to Agilent 5973 mass-selective detector (Agilent, Wokingham, UK). The separation column was a J&W Scientific DB-5MS, 30 m *x* 0.25 mm, 0.25 μm film thickness (J&W Scientific, Milton Keynes, UK). The initial column temperature was 40 ^o^C, this was held for 5 min, then ramped at 8 ^o^C per min to 300 ^o^C and finally held for 15 min before returning to the starting conditions. The nominal flow of the carrier gas (He) was 2.2 ml/min, the purge flow 100 ml/min and the gas pressure 10 psi. Splitless injection was employed with an injector temperature of 250 ^o^C. The mass spectrometer had a source temperature of 200 ^o^C, electrospray ionization was employed and the scan range was 40 – 600 (m/z).

**MALDI-ToF MS, Q-ToF MS and protein identification.** The acidified digests were spotted directly onto a thin layer of matrix on a stainless steel target plate. The matrix consisted of the following: four parts of a saturated solution of α-cyano 4-hydroxycinnamic acid (CCA) in acetone was mixed with one part of a 1:1 mixture of acetone:isopropanol containing 10 mg/ml nitrocellulose. The samples were analysed on a Bruker UltraFlex MALDI-ToF/ToF mass spectrometer (Bruker Daltonics Ltd.). A 50 Hz nitrogen laser was used to desorb/ionise the matrix/analyte material, the acceleration voltage was 25 kV and the ions were detected in positive ion reflectron mode. The samples were externally calibrated against a quadratic calibration curve using a standard mixture of seven peptides ranging in mass from 757.3992 Da to 3147.4700 Da. The obtained mass accuracy was better than 30 ppm. Searches of the generated peptide mass fingerprint (PMF) data were carried out separately using the MASCOT algorithm ([http://www.matrixscience.com](http://www.matrixscience.com/)) against the three available sludge metagenomic amino acid databases (OZ sludge, PHRAP assembly; US sludge, PHRAP assembly; US sludge, JAZZ assembly). The identification of proteins was based on a positive result using MASCOT’s “Probability Based MOWSE Score”. The statistical significance of a MOWSE score depends on the size of the database. Consequently, a MOWSE score of 57 or higher was statistically significant at the 95 % confidence level when searching against the Australian sludge (PHRAP assembly) database, a score of 58 or higher was significant when searching against the US sludge (PHRAP assembly) database, and a score of 55 or higher was significant when searching against the US sludge (JAZZ assembly) database. In addition, no identification was accepted unless at least five peptides representing at least 18 % of the protein sequence were detected in the MALDI-ToF MS peptide mass fingerprint. Proteins not identified from their PMFs, but for which good spectra were obtained, were further analysed by MS/MS on a Q-ToF II mass spectrometer (Micromass) as described earlier (Wilmes and Bond, 2004). Protein identifications were again based on positive results using MASCOT’s “Probability Based MOWSE Score”. For the MS/MS analysis a MOWSE score of 29 or higher was statistically significant at the 95 % confidence level when searching against the Australian sludge (PHRAP assembly) database, a score of 29 or higher was significant when searching against the US sludge (PHRAP assembly) database, and a score of 26 or higher was significant when searching against the US sludge (JAZZ assembly) database. For each positive identification, a minimum of 2 peptides had to be detected.
