# Supplementary material for: Metaproteomics Provides Functional Insight into Activated Sludge Wastewater Treatment
Source: PLoS One. 2008 Mar 12;3(3):e1778. doi: 10.1371/journal.pone.0001778 (PMC2289847; doi:10.1371/journal.pone.0001778)
Supplement: Table S1 — Results of the quantitative FISH analysis (standard deviations in brackets). (0.03 MB DOC) [file pone.0001778.s002.doc]

|  | % of EUBMIX binding cells that gave a positive signal with the specific probe | | | |
| --- | --- | --- | --- | --- |
| Probe | EBPR_28_ | EBPR_42_ | EBPR_55_ | nEBPR_70_ |
| ALF1b | 34 (8.1) | 29 (9.6) | 25 (15.2) | 53 (8.1) |
| BET42a | 63 (14.6) | 68 (2.6) | 72 (9.1) | 41 (6.2) |
| GAM42a | < 1^a^ | < 1^a^ | < 1^a^ | < 1^a^ |
| HGC69a | < 1^a^ | < 1^a^ | < 1^a^ | < 1^a^ |
| CF319a | < 1^a^ | < 1^a^ | < 1^a^ | < 1^a^ |
| PAO651 | 61 (7.3) | 67 (7.8) | 69 (10.8) | < 1^a^ |
| GAOQ431 | < 1^a^ | < 1^a^ | < 1^a^ | < 1^a^ |

^a^Cells were detected with this probe but their numbers were below the detection limit of the quantification method.
