# Supplementary material for: Metaproteomics Provides Functional Insight into Activated Sludge Wastewater Treatment
Source: PLoS One. 2008 Mar 12;3(3):e1778. doi: 10.1371/journal.pone.0001778 (PMC2289847; doi:10.1371/journal.pone.0001778)
Supplement: Table S2 — Summary of the 2D-PAGE analysis of the EBPR matchset (standard deviations in brackets). Indicating numbers of spots detected on individual gels and those matched across the replicate gel sets (n = 3). (0.02 MB DOC) [file pone.0001778.s003.doc]

| Replicate group | Number of spots detected | Number of spots matched |
| --- | --- | --- |
| EBPR_28_ | 1301 (2.6) | 1282 (1.2) |
| EBPR_42_ | 1225 (1.0) | 1207 (1.0) |
| EBPR_55_ | 1621 (2.3) | 1608 (8.6) |
