# Supplementary material for: Metaproteomics Provides Functional Insight into Activated Sludge Wastewater Treatment
Source: PLoS One. 2008 Mar 12;3(3):e1778. doi: 10.1371/journal.pone.0001778 (PMC2289847; doi:10.1371/journal.pone.0001778)
Supplement: Table S3 — Protein identification results obtained using MALDI-ToF MS (spot numbers 1–39), Q-ToF MS/MS (spot numbers 40–46) and MASCOT including additional information (spot numbers refer to those in Fig. 3). (0.88 MB DOC) [file pone.0001778.s004.doc]

| Spot number | Gene object identifier | Protein name [Microbiome] | Scaffold source | Bin | pI | Molecular weight | MASCOT MOWSE score | Sequence coverage / % | Protein expression histogram |
| --- | --- | --- | --- | --- | --- | --- | --- | --- | --- |
| 1 | 2000097400 | F0F1-type ATP synthase, beta subunit [Sludge/US, Phrap Assembly] | sludgePhrap_Contig14464 | not A. phosphatis | 4.8893 | 28561.57 | 106 | 45 | 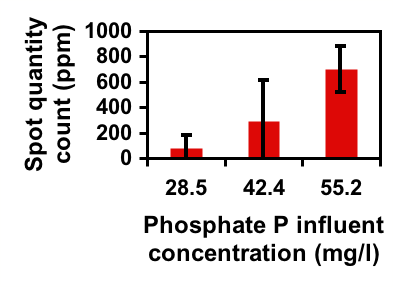 |
| 2 | 2000013070 | Poly(3-hydroxyalkanoate) synthetase [Sludge/US, Phrap Assembly] | sludgePhrap_Contig10729 | not A. phosphatis | 4.6316 | 32221.72 | 138 | 51 | 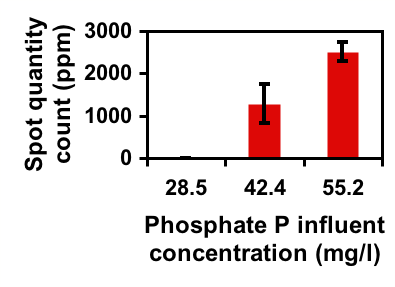 |
| 3 | 2001147280 | Outer membrane protein and related peptidoglycan- associated (lipo)proteins [Sludge/US, Jazz Assembly] | sludgeJazz_scaffold_5382 | not A. phosphatis | 4.6304 | 21158.77 | 97 | 46 | 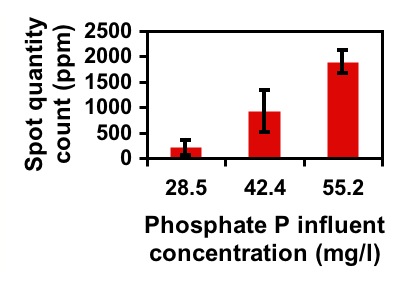 |
| 4 | 2000097400 | F0F1-type ATP synthase, beta subunit [Sludge/US, Phrap Assembly] | sludgePhrap_Contig14464 | not A. phosphatis | 4.8893 | 28561.57 | 64 | 37 | 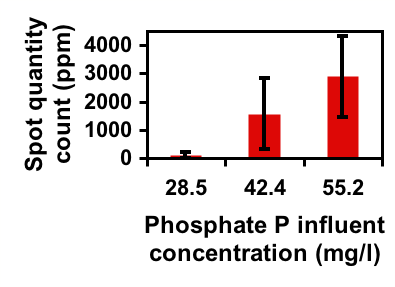 |
| 5 | 2000013070 | Poly(3-hydroxyalkanoate) synthetase [Sludge/US, Phrap Assembly] | sludgePhrap_Contig10729 | not A. phosphatis | 4.6316 | 32221.72 | 85 | 44 | 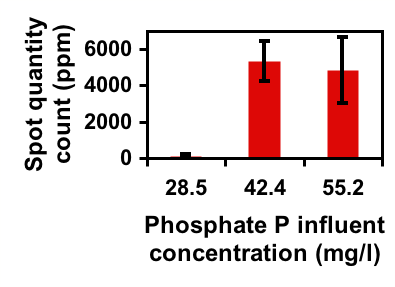 |
| 6 | 2000393020 | F0F1-type ATP synthase, beta subunit [Sludge/OZ, Phrap Assembly] | sludgeOz_Contig11072 | A. phosphatis | 5.1048 | 47660.37 | 173 | 48 | 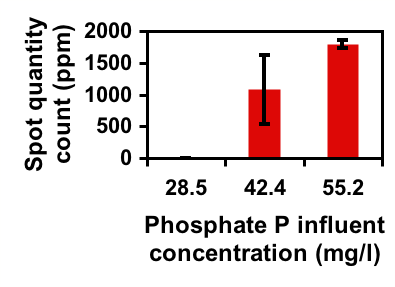 |
| 7 | 2000068750 | Triosephosphate isomerase [Sludge/US, Phrap Assembly] | sludgePhrap_Contig13327 | not A. phosphatis | 5.7883 | 26579.5 | 114 | 44 | 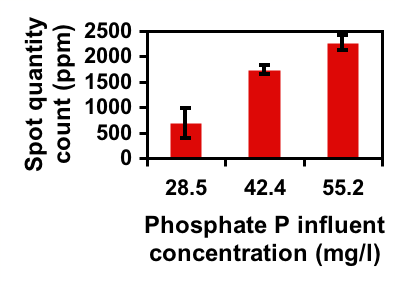 |
| 8 | 2000097400 | F0F1-type ATP synthase, beta subunit [Sludge/US, Phrap Assembly] | sludgePhrap_Contig14464 | not A. phosphatis | 4.8893 | 28561.57 | 194 | 69 | 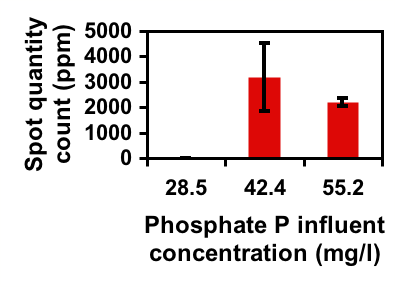 |
| 9 | 2000013070 | Poly(3-hydroxyalkanoate) synthetase [Sludge/US, Phrap Assembly] | sludgePhrap_Contig10729 | not A. phosphatis | 4.6316 | 32221.72 | 68 | 48 | 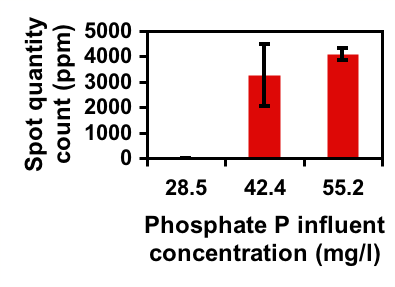 |
| 10 | 2001080810 | Aspartate/tyrosine/aromatic aminotransferase [Sludge/US, Jazz Assembly] | sludgeJazz_scaffold_2344 | not A. phosphatis | 7.0605 | 39054.61 | 72 | 28 | 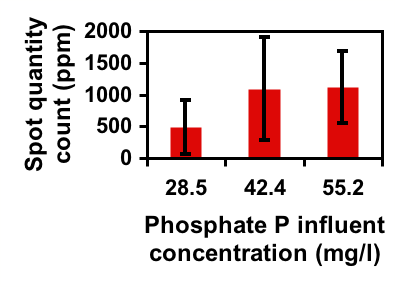 |
| 11 | 2000088860 | F0F1-type ATP synthase, beta subunit [Sludge/US, Phrap Assembly] | sludgePhrap_Contig14141 | not A. phosphatis | 4.739 | 50920.24 | 108 | 34 | 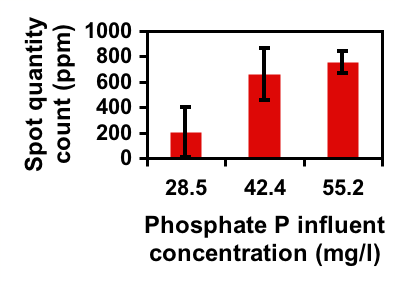 |
| 12 | 2000393020 | F0F1-type ATP synthase, beta subunit [Sludge/OZ, Phrap Assembly] | sludgeOz_Contig11072 | A. phosphatis | 5.1048 | 47660.37 | 110 | 34 | 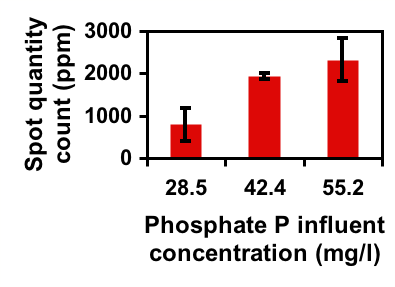 |
| 13 | 2000042020 | GTPases - translation elongation factors [Sludge/US, Phrap Assembly] | sludgePhrap_Contig12119 | not A. phosphatis | 4.6123 | 33081.1 | 86 | 26 | 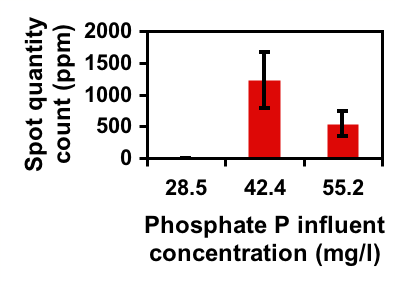 |
| 14 | 2000097400 | F0F1-type ATP synthase, beta subunit [Sludge/US, Phrap Assembly] | sludgePhrap_Contig14464 | not A. phosphatis | 4.8893 | 28561.57 | 157 | 57 | 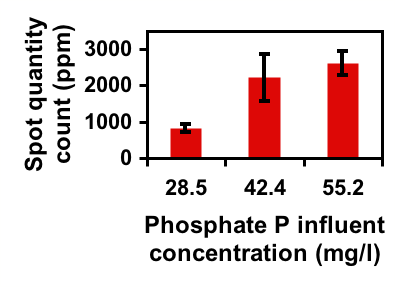 |
| 15 | 2000091820 | Transcription elongation factor [Sludge/US, Phrap Assembly] | sludgePhrap_Contig14251 | not A. phosphatis | 4.2954 | 54543.02 | 137 | 18 | 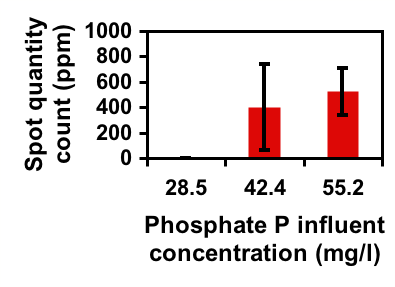 |
| 16 | 2000042020 | GTPases - translation elongation factors [Sludge/US, Phrap Assembly] | sludgePhrap_Contig12119 | not A. phosphatis | 4.6123 | 33081.1 | 76 | 24 | 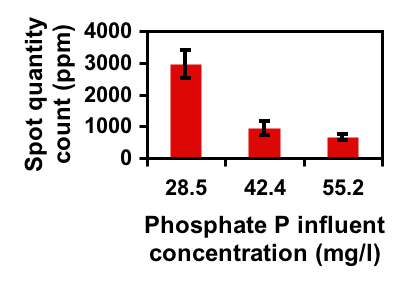 |
| 17 | 2000269280 | Chaperonin GroEL (HSP60 family) [Sludge/US, Phrap Assembly] | sludgePhrap_Contig5612 | not A. phosphatis | 5.0851 | 35862.22 | 110 | 34 | 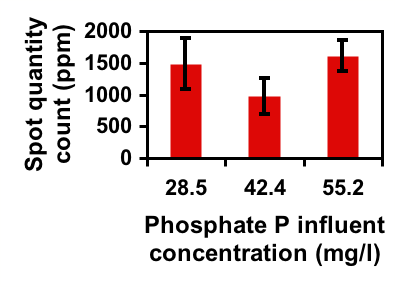 |
| 18 | 2000055830 | Hydroxypyruvate isomerase [Sludge/US, Phrap Assembly] | sludgePhrap_Contig12774 | not A. phosphatis | 5.0321 | 29181.92 | 86 | 34 | 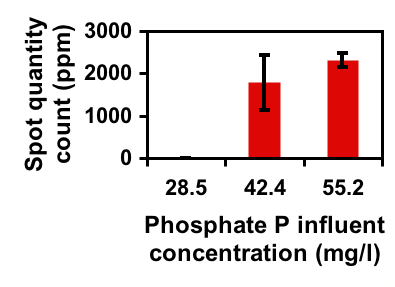 |
| 19 | 2001087690 | Molecular chaperone, HSP90 family [Sludge/US, Jazz Assembly] | sludgeJazz_scaffold_2658 | not A. phosphatis | 4.9897 | 39630.02 | 110 | 34 | 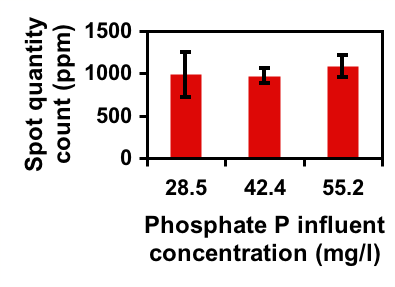 |
| 20 | 2000260810 | Malate synthase [Sludge/US, Phrap Assembly] | sludgePhrap_Contig5136 | not A. phosphatis | 4.6317 | 22905.86 | 105 | 42 | 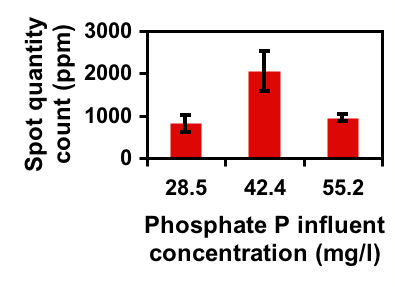 |
| 21 | 2000392840; 2000196160; 2001061540 | Succinate dehydrogenase/fumarate reductase, Fe-S protein subunit [Sludge/OZ, Phrap Assembly; Sludge/US, Phrap Assembly; Sludge/US, Jazz Assembly] | sludgeOz_Contig11070; sludgePhrap_Contig16352; sludgeJazz_scaffold_2 | A. phosphatis; A. phosphatis, Accumulibacter; A. phosphatis, Accumulibacter | 6.4938 | 27253.38 | 82 | 30 | 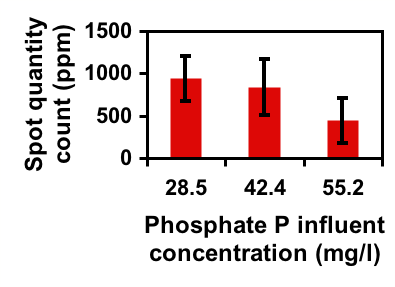 |
| 22 | 2000081840 | Acyl-CoA dehydrogenases [Sludge/US, Phrap Assembly] | sludgePhrap_Contig1388 | not A. phosphatis | 6.7972 | 30880.38 | 68 | 26 | 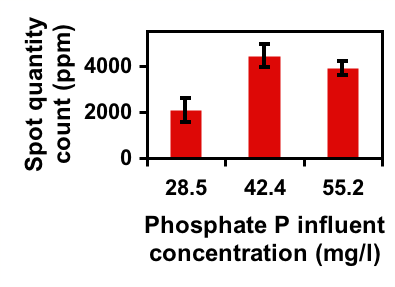 |
| 23 | 2000464240; 2000204490; 2001013540 | Peroxiredoxin [Sludge/OZ, Phrap Assembly; Sludge/US, Phrap Assembly; Sludge/US, Jazz Assembly] | sludgeOz_Contig11604, sludgePhrap_Contig16364 and sludgeJazz_scaffold_1 | A. phosphatis, Accumulibacter; A. phosphatis, Accumulibacter; A.phosphatis, Accumulibacter | 5.2572; 5.2572; 5.2572 | 20487.36; 20469.32; 20469.32 | 73 | 33 | 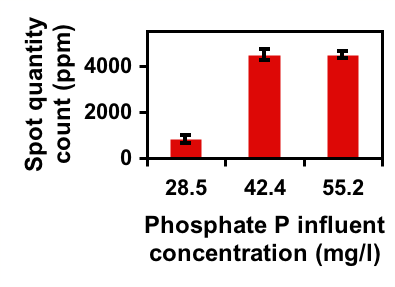 |
| 24 | 2000411770; 2000163970; 2001002930 | GTPases - translation elongation factors [Sludge/OZ, Phrap Assembly; Sludge/US, Phrap Assembly; Sludge/US, Jazz Assembly] | sludgeOz_Contig11307, sludgePhrap_Contig16233 and sludgeJazz_scaffold_1 | A. phosphatis; A. phosphatis; A. phosphatis, Accumulibacter | 5.8095; 5.8095; 5.8095 | 42918.21; 42948.24; 42990.32 | 110 | 34 | 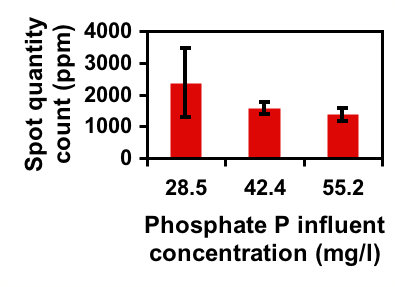 |
| 25 | 2000411770; 2000163970; 2001002930 | GTPases - translation elongation factors [Sludge/OZ, Phrap Assembly, Sludge/US, Phrap Assembly; Sludge/US, Jazz Assembly] | sludgeOz_Contig11307, sludgePhrap_Contig16233 and sludgeJazz_scaffold_1 | A. phosphatis; A. phosphatis; A. phosphatis, Accumulibacter | 5.8095; 5.8095; 5.8095 | 42918.21; 42948.24; 42990.32 | 108 | 31 | 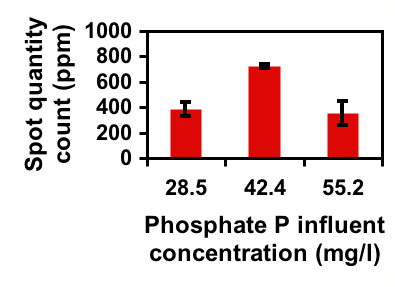 |
| 26 | 2001037840 | Uncharacterized NAD(FAD)-dependent dehydrogenases [Sludge/US, Jazz Assembly] | sludgeJazz_scaffold_1198 | not A. phosphatis | 8.4064 | 54026.85 | 67 | 22 | 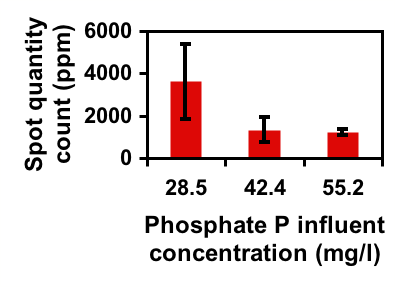 |
| 27 | 2000013070 | Poly(3-hydroxyalkanoate) synthetase [Sludge/US, Phrap Assembly] | sludgePhrap_Contig10729 | not A. phosphatis | 4.6316 | 32221.72 | 151 | 33 | 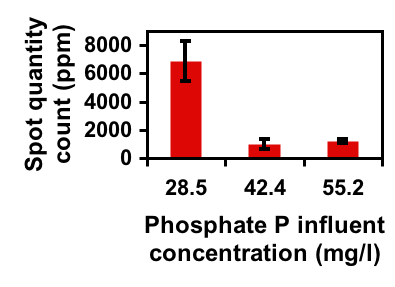 |
| 28 | 2000013070 | Poly(3-hydroxyalkanoate) synthetase [Sludge/US, Phrap Assembly] | sludgePhrap_Contig10729 | not A. phosphatis | 4.6316 | 32221.72 | 136 | 51 | 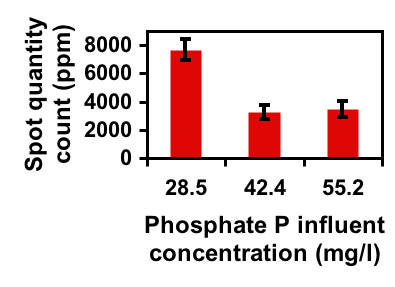 |
| 29 | 2000142340 | Molecular chaperone, HSP90 family [Sludge/US, Phrap Assembly] | sludgePhrap_Contig15850 | not A. phosphatis | 5.0524 | 71178.15 | 159 | 24 | 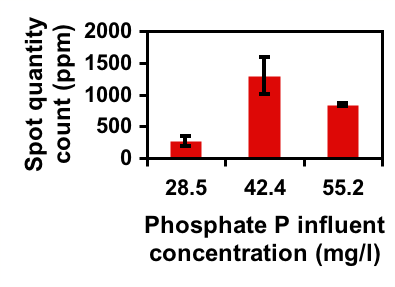 |
| 30 | 2000407730 | Thiol-disulfide isomerase and thioredoxins [Sludge/OZ, Phrap Assembly] | sludgeOz_Contig11264 | A. phosphatis, Accumulibacter | 8.6005 | 36526.48 | 58 | 26 | 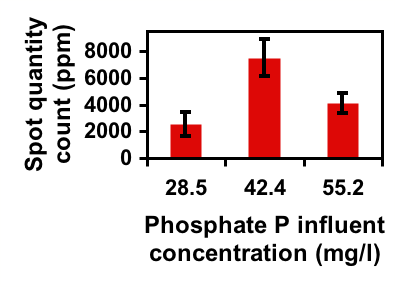 |
| 31 | 2000117530; 2001032100 | 2-keto-4-pentenoate hydratase/2-oxohepta-3-ene-1, 7-dioic acid hydratase (catechol pathway) [Sludge/US, Phrap Assembly; Sludge/US, Jazz Assembly] | sludgePhrap_Contig15158; sludgeJazz_scaffold_1030 | not A. phosphatis, not A. phosphatis | 5.6127; 5.6127 | 24251.54; 24251.54 | 71 | 36 | 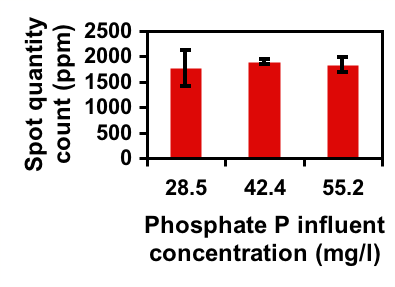 |
| 32 | 2000055360 | Peroxiredoxin [Sludge/US, Phrap Assembly] | sludgePhrap_Contig12752 | not A. phosphatis | 7.4351 | 17130.41 | 66 | 34 | 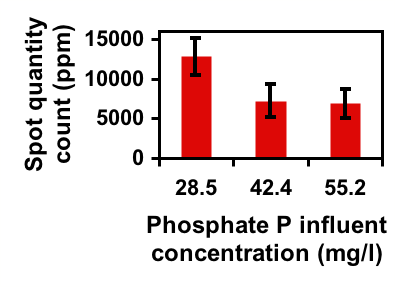 |
| 33 | 2001154530 | Phosphoenolpyruvate synthase/pyruvate phosphate dikinase [Sludge/US, Jazz Assembly] | sludgeJazz_scaffold_646 | not A. phosphatis | 6.7228 | 33081.04 | 104 | 33 | 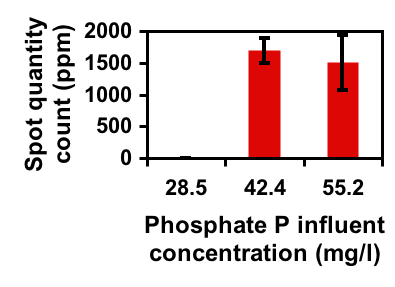 |
| 34 | 2000171550 | hypothetical protein [Sludge/US, Phrap Assembly] | sludgePhrap_Contig16278 | A. phosphatis, Accumulibacter | 9.7332 | 24691.13 | 70 | 36 | 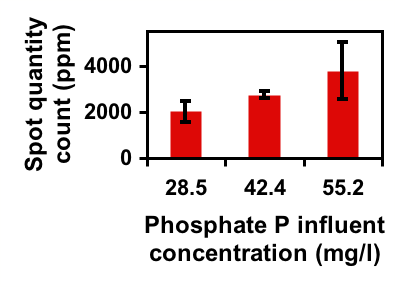 |
| 35 | 2000274860 | hypothetical protein [Sludge/US, Phrap Assembly] | sludgePhrap_Contig5914 | not A. phosphatis | 6.9602 | 28580.48 | 74 | 33 | 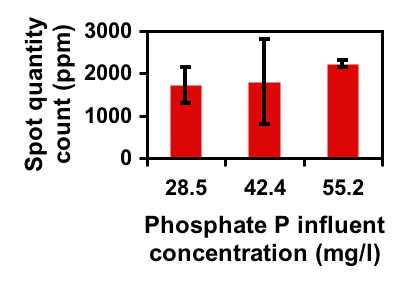 |
| 36 | 2000274860; 2000126150 | hypothetical protein [Sludge/US, Phrap Assembly]; ABC-type phosphate transport system, periplasmic component [Sludge/US, Phrap Assembly] | sludgePhrap_Contig5914; sludgePhrap_Contig15425 | not A. phosphatis; Betaproteobacteria (<85),  not A. phosphatis | 6.9602; 9.6479 | 28580.48; 19377.06 | 66 | 32 | 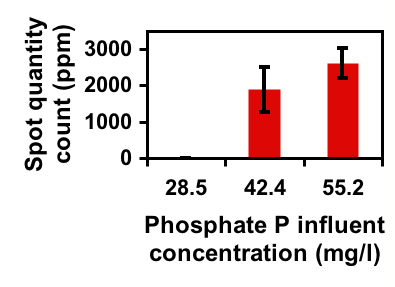 |
| 37 | 2001139260 | ADP-glucose pyrophosphorylase [Sludge/US, Jazz Assembly] | sludgeJazz_scaffold_4901 | not A. phosphatis | 6.7278 | 28128.69 | 79 | 34 | 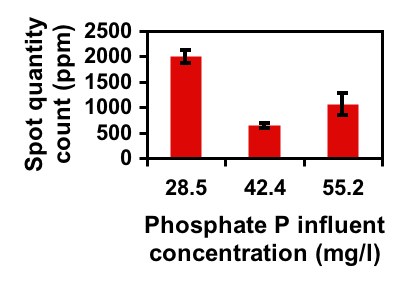 |
| 38 | 2000072380 | Phosphoenolpyruvate synthase/pyruvate phosphate dikinase [Sludge/US, Phrap Assembly] | sludgePhrap_Contig13471 | not A. phosphatis | 7.1147 | 51345.3 | 96 | 23 | 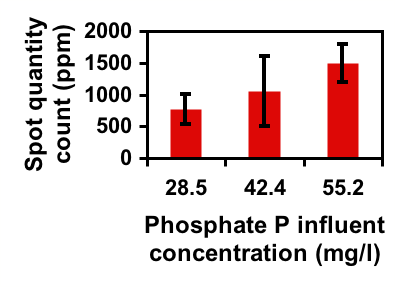 |
| 39 | 2000281060 | Biotin carboxylase [Sludge/US, Phrap Assembly] | sludgePhrap_Contig6268 | not A. phosphatis | 4.6091 | 16815.8 | 183 | 37 | 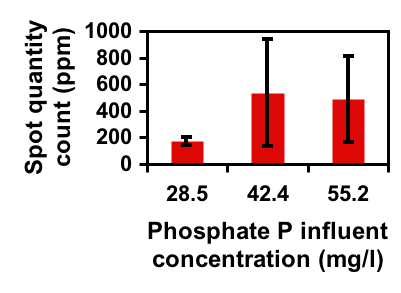 |
| 40 | 2000422390; 2001070410; 2000162760 | Enoyl-CoA hydratase [Sludge/OZ, Phrap Assembly; Sludge/US, Jazz Assembly; Sludge/US, Phrap Assembly] | sludgeOz_Contig11396; sludgeJazz_scaffold_2; sludgePhrap_Contig16221 | A.phosphatis, Accumulibacter; A. phosphatis, Accumulibacter; A. phosphatis, Accumulibacter | 5.4806 | 27873.13 | 152 | 12 | 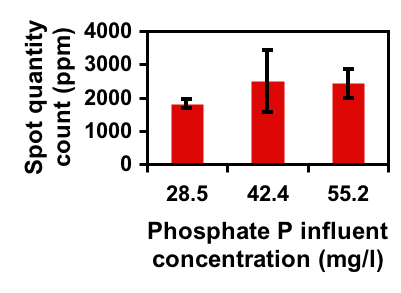 |
| 41 | 2001139020; 2000322220 | Acetyl-CoA acetyltransferase [Sludge/US, Jazz Assembly; Sludge/US, Phrap Assembly] | sludgeJazz_scaffold_4895; sludgePhrap_Contig8627 | not A. phosphatis; not A. phosphatis | 7.9466 | 13704.9 | 43 | 29 | 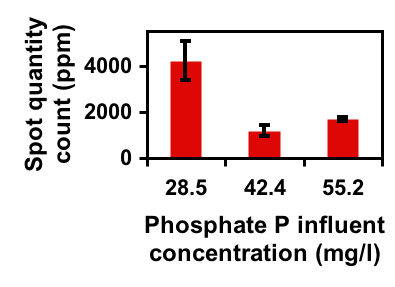 |
| 42 | 2000615360 | Topoisomerase IA [Sludge/OZ, Phrap Assembly] | sludgeOz_Contig8634 | not A. phosphatis | 7.743 | 65352.58 | 33 | 32 | 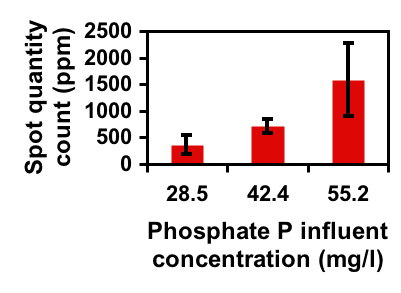 |
| 43 | 2001103000 | Citrate synthase [Sludge/US, Jazz Assembly] | sludgeJazz_scaffold_3140 | not A. phosphatis | 6.2376 | 31949.54 | 28 | 7 | 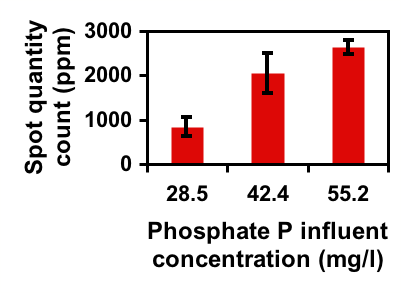 |
| 44 | 2000471430; 2001005820; 2000184460 | Acyl-coenzyme A synthetases/AMP-(fatty) acid liga ses [Sludge/OZ Phrap Assembly; Sludge/US, Jazz Assembly; Sludge/US, Phrap Assembly] | sludgeOz_Contig11621; sludgeJazz_scaffold_1; sludgePhrap_Contig16325 | A. phosphatis, Accumulibacter; A. phosphatis, Accumulibacter; A. phosphatis, Accumulibacter | 6.2542 | 71213.24 | 57 | 4 | 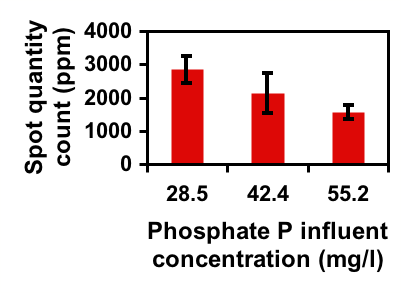 |
| 45 | 2000460860; 2001011240; 2000185640 | Glutamyl- and glutaminyl-tRNA synthetases [Sludge/OZ, Phrap Assembly; Sludge/US, Jazz Assembly; Sludge/US, Phrap Assembly] | sludgeOz_Contig11592; sludgeJazz_scaffold_1; sludgePhrap_Contig16328 | A. phosphatis; A. phosphatis, Accumulibacter; A. phosphatis, Accumulibacter | 6.0106 | 69441.75 | 34 | 7 | 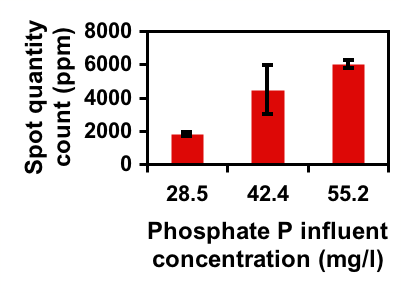 |
| 46 | 2001046750; 2000025640 | hypothetical protein [Sludge/US, Jazz Assembly; Sludge/US, Phrap Assembly] | sludgeJazz_scaffold_152; sludgePhrap_Contig11326 | Accumulibacter, not A. phosphatis; not A. phosphatis | 5.7818 | 14076.7 | 113 | 30 | 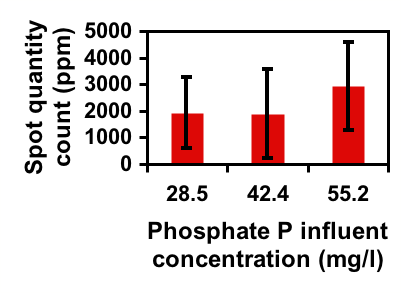 |
