# Supplementary figures and images for: Metaproteomics Provides Functional Insight into Activated Sludge Wastewater Treatment
Source: PLoS One. 2008 Mar 12;3(3):e1778. doi: 10.1371/journal.pone.0001778 (PMC2289847; doi:10.1371/journal.pone.0001778)

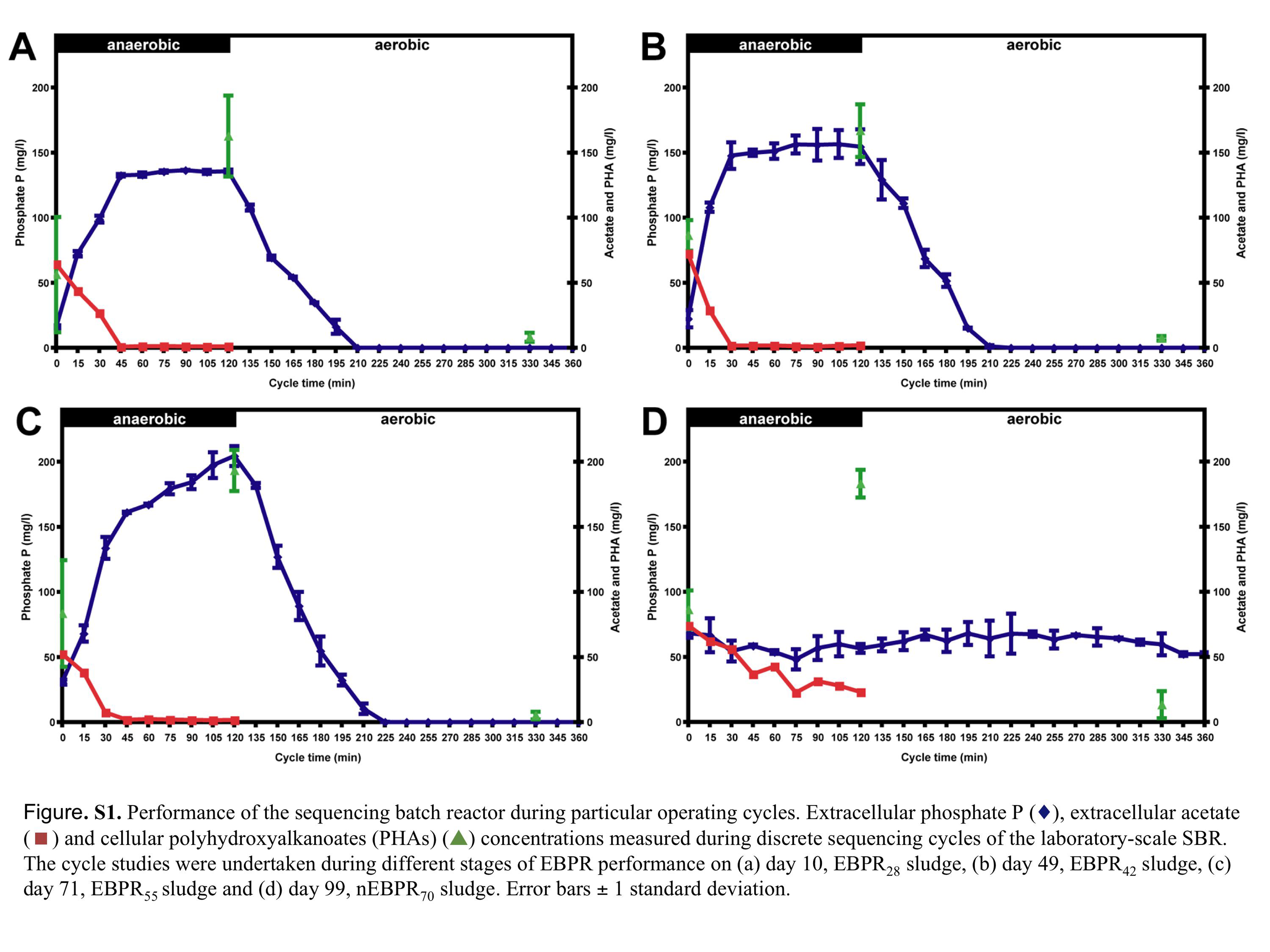

Supplement: Figure S1 — (4.07 MB TIF) [file pone.0001778.s005.tif]
